# Oral Complementary Medicine Use among People with Inflammatory Arthritis: An Australian Rheumatology Association Database Analysis

**DOI:** 10.1155/2020/6542965

**Published:** 2020-06-05

**Authors:** Ashley Fletcher, Marissa Lassere, Lyn March, Catherine Hill, Graeme Carroll, Claire Barrett, Rachelle Buchbinder

**Affiliations:** ^1^Monash Department of Clinical Epidemiology, Cabrini Institute, Melbourne, Australia; ^2^Department of Epidemiology and Preventive Medicine, School of Public Health and Preventive Medicine, Monash University, Melbourne, Australia; ^3^St George Hospital, NSW, Australia; ^4^Florance and Cope Professorial Department of Rheumatology, Royal North Shore Hospital, Institute of Bone and Joint Research, University of Sydney, Australia; ^5^The Queen Elizabeth and Royal Adelaide Hospitals, Adelaide, University of Adelaide, Australia; ^6^Department of Rheumatology, Fiona Stanley Hospital, Australia; ^7^Redcliffe Hospital, University of Queensland, Australia

## Abstract

**Objectives:**

To describe oral complementary medicine (CM) use in people with inflammatory arthritis, associations with use, and changes in use over time.

**Methods:**

Demographic, clinical, and patient-reported outcome data from 5,630 participants with rheumatoid arthritis (RA), ankylosing spondylitis (AS), psoriatic arthritis (PsA), and juvenile idiopathic arthritis (JIA) were extracted from the Australian Rheumatology Association Database (ARAD), a national observational database. CM use at entry into ARAD was ascertained for participants recruited between 2002 and 2018. CM was categorised according to the NIH/Cochrane schema (fatty acids, herbs, or supplements). Logistic regression was used to assess associations between demographic characteristics and CM use. Change in CM use between 2006 and 2016 was investigated using a nonparametric test for trend of rate by year.

**Results:**

2,156 (38.3%) ARAD participants were taking CM at enrolment (RA: 1,502/3,960 (37.9%), AS: 281/736 (38.2%), PsA: 334/749 (44.6%), and JIA: 39/185 (21.1%)). CM use was more prevalent in women (OR 1.3; 95% CI: 1.13-1.50), those with tertiary education (OR 1.32; 95% CI: 1.13-1.55), private health insurance (OR 1.26; (95% CI: 1.10-1.44), drinking alcohol sometimes (OR 1.22; 95% CI: 1.05-1.43), poorer function (HAQ) (OR 1.13; 95% CI: 1.02-1.24), use of NSAID (OR 1.32; 95% CI 1.17-1.50), weak (OR 1.21; 95% CI 1.05-1.41) but not strong opioids, and less prevalent in current smokers (OR 0.76; 95%: CI 0.63-0.91). CM use was not associated with pain, disease activity, or quality of life. The most common CMs were fish oils (*N* = 1,489 users) followed by glucosamine (*N* = 605). Both declined in use over time between 2006 and 2016 (27.5% to 21.4%, trend *p* = 0.85 and 15.5% to 6.4%, trend *p* < 0.01), respectively.

**Conclusion:**

Oral CM use is common among Australians with inflammatory arthritis. Its use is greater among women and those with tertiary education. Fish oil and glucosamine, the most common CMs, both declined in use over time.

## 1. Introduction

Inflammatory arthritis comprises a range of conditions that affect joints and other tissues. These conditions include rheumatoid arthritis (RA), psoriatic arthritis (PsA), ankylosing spondylitis (AS), and juvenile idiopathic arthritis (JIA). In Australia, the prevalence of RA ranges between 0.46% from a systematic review that included doctor-diagnosed RA only [1] to 1.9% according to the 2017-18 National Health Survey which relies solely upon self-report [2]. Up to 0.4% of school children are reported to have JIA [3], and AS and PsA have a reported prevalence of 0.25% and 0.19%, respectively [4].

Treatment for inflammatory arthritis depends on the type of disease, its symptoms, and severity. When indicated, conventional disease-modifying antirheumatic drugs (csDMARDs) and biologic/targeted synthetic DMARDs (b/tsDMARDs) are prescribed to slow or stop the progression of the disease and limit or prevent joint damage [5]. Nonsteroidal anti-inflammatory drugs (NSAIDs) and glucocorticoids are also sometimes indicated to manage pain and inflammation and may also have disease-modifying effects for some types of inflammatory arthritis [6, 7].

Complementary medicine (CM) is a broad term that encompasses a range of therapies, usually natural products, and also mind and body practices. Natural products include fatty acids (e.g., fish oil, New Zealand (NZ) green-lipped mussels, and evening primrose oil), herbs (e.g., ginger, celery, and Chinese herbs), vitamins (e.g., vitamin C) and multivitamins, minerals (e.g., magnesium and zinc), complex compounds (e.g., glucosamine and chondroitin), and probiotics, all commonly available as dietary supplements [8]. CM may or may not be recommended by health professionals and may be used alone or in combination with conventional medicine. Different forms of CM are frequently used by patients with chronic diseases including inflammatory arthritis [9].

Two separate population-based studies performed in Australia reported that over a third of those with arthritis use oral CM [9, 10]. A national health survey reported that 36.1% of 25,906 adults with self-reported arthritis use oral CM [10], while 38.4% of 3,161 participants in the North West Adelaide Health Study with self-reported doctor-diagnosed arthritis reported CM use [9]. Clinic-based estimates vary widely depending upon the setting. For example, 23% were found to be using oral CM in a hospital-based early inflammatory arthritis clinic in Singapore [11], while the rate has been estimated to vary from 35% to 63% in RA clinics in Japan [12] and Australia [13], respectively. In an American general practice study 26.6% of patients with RA were taking supplements, 20.8% were taking vitamins and minerals, and 10.1% were taking herbs [14].

Studies have also shown that oral CM use is common in AS. In a cross-sectional study of 75 attendees of an Australian AS clinic, 72.1% were taking a dietary CM at the time of the study [15]. The prevalence of use is reportedly lower in JIA; 17% of 235 attendees of a Canadian arthritis clinic were reported to be using dietary changes or supplements [16], while 29% of 134 patients in US paediatric rheumatology clinics report current herbal medicine use [17]. No studies have looked at the prevalence of dietary CM use in people with PsA.

The aim of this study was to describe the use of oral CM among people with RA, PsA, AS, and JIA contributing to the Australian Rheumatology Association Database (ARAD), a large national prospective observational registry. Secondary aims were to determine any associations with oral CM use and the demographic and clinical characteristics of ARAD participants and investigate trends in types of oral CM used over time.

## 2. Methods

### 2.1. Australian Rheumatology Association Database (ARAD)

ARAD is a voluntary national registry that collects longitudinal health outcome data from people with inflammatory arthritis. It includes participants with RA, PsA, AS, and JIA [18]. Most participants enroll when they commence a b/tsDMARD. Enrolment is also encouraged for those not starting b/tsDMARDs but active targeting of this group has only occurred on an ad hoc basis. Based on residential postcode, demographic and clinical characteristics participants appear to be nationally representative, with rheumatologists from all states and territories having contributed patients [19]. ARAD has received ethical approval from committees and organisations across Australia. All participants provide written permission to be contacted by ARAD investigators and informed consent to participate in the registry as well as have their data linked with other national databases including state and territory cancer registries, death registry, and the pharmaceutical and medical benefits schemes.

As described previously [18–20], at enrolment, details of diagnosis, disease status, and b/tsDMARD prescribed (if applicable) are obtained from the treating rheumatologist. All ARAD participants complete detailed entry and 6- to 12-monthly follow-up questionnaires (paper-based or online). Data collected from the participants include demographic and socioeconomic details, disease duration and severity, self-reported past, and current medical history including malignancies and other chronic conditions, use of antirheumatic drugs with the date commenced and ceased, other medications including CM, and smoking and alcohol history. Details concerning the level of pain experienced (0 to 100 scale where 0 is no pain and 100 is as bad as it could be) and overall arthritis activity (0 to 100 scale where 0 is none and 100 is extreme) in the preceding week are also ascertained. Arthritis-specific disability is assessed by the Health Assessment Questionnaire (HAQ) (0 to 3 where higher scores indicate greater disability) [21], while quality of life is measured with the Assessment of Quality of Life (AQoL) (0 to 1 where higher score indicates better quality of life) [22], European Quality of Life (EuroQoL or EQ-5D (UK)) (0 to 1 where 0 is worst imaginable health state and 1 is the best imaginable health state) [23], and the Medical Outcomes Survey Short Form (SF-36) (0 to 100 where a higher score indicates better health) [24].

In the event of missing or ambiguous data, ARAD staff contact the participant, the rheumatologist, and/or other treating doctor to verify the data. For the purpose of this study, ARAD participants were eligible if they enrolled between 1 September 2001 and 31 May 2018.

### 2.2. Complementary Medication

Self-reported use of oral CM therapy for arthritis treatment is collected at each questionnaire. Current use of ginger, St John's wort, celery seed, pennywort, Chinese herbs, glucosamine, fish oil, homeopathic remedies, NZ green-lipped mussels, kelp, and shark fin are asked about specifically. The use of other CMs is obtained from two free text fields.

For the purpose of this study, we grouped CMs, as defined by NIH [25] and Cochrane [8], into three categories: fatty acids, herbal medicine, and supplements. Fatty acids are animal and vegetable oils that contain omega-3 (e.g., fish oil and evening primrose oil); herbal medicines are plant preparations (e.g., ginger and Chinese herbs); and supplements are vitamins, minerals (e.g., magnesium and zinc), and complex compounds such as amino sugars (e.g., glucosamine and chondroitin). We excluded calcium, iron, and vitamin D supplements as they are not specific for inflammatory arthritis and/or could have been prescribed for other reasons, while folic acid use was excluded as this is prescribed with methotrexate.

### 2.3. Statistical Analyses

Selected baseline characteristics of the ARAD cohort were assessed by disease type using descriptive statistics, frequency, and chi-squared for categorical variables, ANOVA, or Kruskal-Wallis tests for continuous variables. Analyses were performed with and without JIA to assess if the JIA population influenced the results. Baseline was defined as the entry date into ARAD (first questionnaire completed). Baseline responses were compared for CM users and those not taking any CMs. Logistic regression models were used to assess associations between baseline demographic characteristics and CM use. Multivariable modelling was performed to ascertain independent predictors of CM use.

Employment was coded as working, not working, and permanently unable to work due to illness. Not working includes home duties, student, retired, and others from a free text field that includes casual work, volunteer work, and carers. The Physical Component Summary (PCS) and Mental Component Summary (MCS) scores of the SF-36 were calculated using standard algorithms based on population norms for Australia. For socioeconomic status (SES), we assigned an Australian Bureau of Statistics (ABS) Socio-Economic Indexes for Areas (SEIFA) score. We coded SES according to the Index of Relative Socio-economic Advantage and Disadvantage (IRSAD). The location was based on the participant's baseline address and classified at Statistical Areas Level 1 (SA1). SEIFA 1 is the lowest quintile with relatively greater disadvantage and a lack of advantage in general; SEIFA 5 is the highest quintile with a relative lack of disadvantage and greater advantage in general.

Potential explanatory variables included in the model were age, sex, disease duration, HAQ, AQoL, EQ-5D (UK), pain level in the last week, overall arthritis activity in the last week, education (tertiary/secondary/did not completed secondary), employment (working, not working/home duties/student/retired/other, or permanently unable/ill), SES, current private health insurance, current smoking status, alcohol status (never, sometimes, or every day), current b/tsDMARD/prednisolone/methotrexate status, opioid, and NSAID use. Opioids were classified as high (morphine, oxycodone, hydromorphone, methadone, buprenorphine, fentanyl, sufentanil, tapentadol, and tramadol), and low (aspirin/codeine, paracetamol/codeine, dextropropoxyphene) potency.

Variables were retained in the multivariable model if they remained significant at *p* = 0.05. Results are expressed as odds ratios (ORs) and 95% confidence interval (CI).

CM use between 2006 and 2016, the period with high numbers at baseline, is represented graphically and statistically using a nonparametric test for trend of rate by year. All analyses were performed using Stata 14.

## 3. Results

There were 5,630 ARAD participants included in this analysis (RA: *N* = 3,960 (70.3%); PsA: *N* = 749 (13.3%), AS: *N* = 736 (13.1%), JIA: *N* = 185 (3.3%)) (Table 1). Overall participants were predominantly Caucasian (94.1%). There were significant differences between the disease populations. Compared with RA participants, other disease groups were significantly younger (*p* < 0.01 for each comparison); a higher proportion of females had RA, JIA, and PsA, but a lower proportion had AS; and disease duration was shorter on average for JIA than other diagnoses. In addition, a lower proportion of RA participants had completed tertiary education and they were less likely to be in current employment and have private health insurance in comparison to PsA and AS participants, while almost three-quarters of JIA participants were still students.

RA participants also had higher pain levels and greater disability and poorer health-related quality of life compared with the other disease groups, as indicated by higher mean HAQ, lower mean SF-36 (PCS and MCS scores), and AQoL scores. The majority of participants across all diagnosis categories were taking b/tsDMARDs at baseline (55.6% overall), while current methotrexate and prednisolone use was higher among participants with RA.

### 3.1. Complementary Medicine Use

Overall, 2156 (38.3%) of the cohort were taking at least one CM at baseline (Table 2). Homeopathic remedies (*n* = 47) were included in the overall analysis, but not grouped by category because they had many ingredients across categories. Participants with PsA had the greatest use (44.6%), followed by AS (38.2%), RA (37.9%), and JIA (21.1%). Among those with CM use, 57.4% were taking one, 27.7% were taking two, and 14.9% were taking three or more CMs.

Table 2 shows the type of CM in use across all ARAD participants at baseline. The most common category of CM was fatty acids (*N* = 1,564), most commonly fish oil (*n* = 1,489, 95.2% of fatty acids). There were 808 participants using supplements, most commonly glucosamine (*n* = 605, 74.9% of supplements), and 296 participants using herbal medicines, most commonly ginger (*n* = 148, 50.0% of herbs).

Table 3 presents the results of the univariate and multivariable analysis investigating differences between CM users and nonusers. In the univariate analysis, CM use was associated with having PsA, older age, being female, having a tertiary education, higher SES, having private health insurance, better self-reported function (lower HAQ), and worse health-related quality of life (higher AQoL and EQ-5D) but showed no difference in SF-36 physical and mental function scores, level of pain or overall arthritis activity in the last week. CM use was also more likely in nonsmokers and having alcohol sometimes and every day compared with never. CM use was also associated with NSAID and both low and high potency opioid use. CM use was not related to employment.

Variables that remained significant in the multivariable analysis included being female (OR 1.30; 95% CI: 1.13-1.50), tertiary education (OR 1.32; 95% CI: 1.13-1.55), private health insurance (OR 1.26; (95% CI: 1.10-1.44), drinking alcohol sometimes (OR 1.22; 95% CI: 1.05-1.43), poorer function (higher HAQ) (OR 1.13; 95% CI: 1.02-1.24), and concurrent use of NSAIDs (OR 1.32; 95% CI 1.17-1.50) and weak (OR 1.21; 95% CI 1.05-1.41) but not strong opioids. CM use was less prevalent in current smokers (OR 0.76; 95%: CI 0.63-0.91) and no longer associated with pain or self-reported arthritis activity in the past week or quality of life.

### 3.2. Changes in Complementary Medication Use over Time

The proportion of participants with CM use did not appreciably change over the 6 and 12-month follow-up from baseline. Compared to baseline (38.3%), at the six-month follow-up questionnaire, out of 4,077/5630 (72.4%) participants with data, 37.9% were taking at least one CM. This was similar to the 38.2% taking at least one CM at 12 months (data for 3,901/5630 (69.3%) participants) (data not shown).

**Figure 1:**
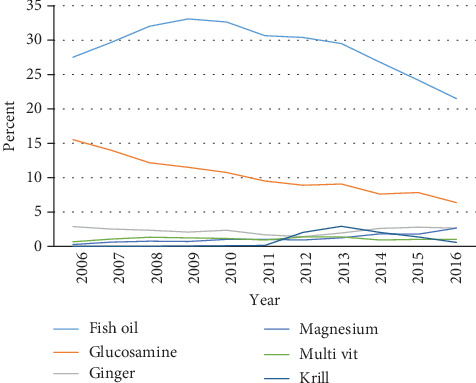
Trend in prevalence of common complementary medicines between 2006 and 2016 using all questionnaires.

Figure 1 shows CM use over time for the most common CMs recorded at any questionnaire between 2006 and 2016. Fish oil, while still the most common form of CM used in this cohort, declined in use over time from 27.5% in 2006 to 21.4% in 2016 although the trend was nonsignificant (*p* = 0.85). Glucosamine use also declined over this period (15.5% to 6.4%, trend *p* < 0.01). Ginger remained stable over time (2.9% to 2.6%, trend *p* = 0.37), calcium decreased (2.1% to 1.0%, trend *p* = 0.71), while magnesium (0.3% to 2.7%, trend *p* = 0.02) and multivitamins (0.5% to 0.9%, trend *p* = 0.97) both increased. Krill oil started to be used in 2009, peaked in 2013 (3.0%), and has been decreasing since then (0.8% in 2016). Other CMs listed in Table 1 were used by a lower proportion of participants but those with significant changes, in the test for trend, between 2006 and 2016 were celery seed (0.9% to 0.4%), shark fin (1.3% to 0.3%), pennywort (0.4% to 0.04%), evening primrose oil (0.8% to 0.2%), turmeric (0.0% to 1.5%), rosehip (0.2% to 0.1%, but peaked in 2014 at 0.5%), and probiotic (0.0% to 0.9%).

## 4. Discussion

CM use is common among people with inflammatory arthritis in Australia as evident by the 38.3% of ARAD participants taking at least one CM at entry into the database. This is consistent with the prevalence rates reported in the 2004-05 Australian National Health Survey (NHS) [9] and the North West Adelaide Health Study (NWAHS) cohort in 2004-05 [10]. Use among JIA participants was much lower compared to the other disease groups. Of those taking at least one CM, just under half were taking two or more. The most common CMs were fatty acids, predominantly fish oil, and supplements, most commonly glucosamine. However, the use of both of these CMs declined over the decade from 2006 to 2016.

Similar to other studies, we found that CM use was associated with being female and being tertiary educated [26–28]. Other associations which remained in the multivariable analysis included having private health insurance, not currently smoking, consuming alcohol sometimes (rather than never), having poorer function, and current low potency opioid and NSAID use. We did not investigate the association with place of residence although another Australian study found an association with living in rural and remote areas [29].

Our results indicate that the use of particular types of CM fluctuates over time. The two most popular CMs, fish oils, and glucosamine have been declining in use over time, while supplements (mainly vitamins) have been increasing over time. An Australian study of oral CM use in the general population of South Australia at three time points (1993, 2000 and 2004) reported that oral CM use rose from 48.5% to 52.2% between 1993 and 2004 [27]. Reasons for use were for general health (70%) followed by for muscle, bones, or joints (21%), for the immune system (18.2%), for nerves or stress (13.0%), and for blood or circulation (9.5%). In an Australian population-matched cross-sectional survey of 2,025 Australian adults in 2019 the use of CM was found to be 50.3% [30].

High quality and robust evidence to support the use of various oral CMs for RA, PsA, AS, and JIA is lacking. A 2017 systematic review based upon 22 trials found moderate-quality evidence for a small favourable effect (SMD -0.21 (95% CI -0.42 to -0.00) translating to <8% improvement) for marine oil supplements in RA [31]. However, the effects varied depending upon the type of marine oil, dose, and ratio of eicosapentaenoic acid (EPA) and docosahexaenoic acid (DHA). A Cochrane review of herbal therapy for RA, last updated in 2011, found moderate-quality evidence that oils containing gamma linolenic acid (GLA) (evening primrose, borage, or blackcurrant seed oil) may also provide symptom benefit in RA [32].

A strength of our study is that ARAD collects longitudinal data enabling us to determine whether CM use has changed over time. Our study is also the first to provide a population-based estimate of the prevalence of CM use among people with PsA as well as add to the limited data available for CM use in AS [15] and JIA [16, 17]. A generally high number of ARAD participants complete questionnaires each year, 12.7% only complete one questionnaire, 87.3% complete 2 questionnaires, and 78.9% complete 3 questionnaires. Loss to follow-up may be a potential source of bias for our trend analyses although those that filled in 6 and 12-month questionnaires had similar rates of CM use (37.9% and 38.2%, respectively) compared to baseline (38.3%). Another strength was the size of our cohort allowing us to investigate the associations of CM use across a large number of participants with diverse demographic, disease, and treatment characteristics.

Potential limitations include the fact that participation in ARAD is voluntary and therefore our results may not be generalisable to the Australian population overall or by inflammatory arthritis type. However, overall CM use in ARAD was similar to use in people with arthritis found in two Australian population-based studies, which is reassuring and strengthens the case for validity. Although we specifically ask ARAD participants to specify CMs taken for arthritis, we cannot be certain that this is the case. We also have no data concerning the amount or dose of the CMs used. Our study only investigated oral forms of CM and did not consider other CM categories such as alternative medical systems, energy therapies, manipulative and body-based methods, mind-body interventions, or other natural product-based therapies.

## 5. Conclusion

Based upon the findings from ARAD, a large national prospective registry, oral CM use is common among Australians with all types of inflammatory arthritis including JIA. Over a third of people with inflammatory arthritis were taking at least one CM at the time they entered ARAD and this was most common in people with PsA. The observation that many patients with rheumatic diseases use CM in Australia as a complement to their prescribed medications is important information for clinicians. Obtaining details of patients' CM use is important particularly in considering adverse events and possible interactions with prescribed medicines. CM use was greater among women and those who are better educated. Trends in which types of CM are used changes over time. Fish oil and glucosamine were the most commonly used CM but they both declined in use over the period of study.

## Figures and Tables

**Table 1:** Demographic and clinical details of Australian Rheumatology Association Database (ARAD) participants by disease group at baseline and their complementary medicine use, *N* = 5,630.

Variable	Rheumatoid arthritis (*N* = 3,960)	Psoriatic arthritis (*N* = 749)	Ankylosing spondylitis (*N* = 736)	Juvenile arthritis (*N* = 185)
	Mean (SD)	Mean (SD)	Mean (SD)	Mean (SD)
Mean (SD) age (years)	56.8 (13.0)	52.3 (12.3)	45.8 (12.7)	13.4 (6.9)
Disease duration (years)	12.7 (10.8)	10.7 (9.3)	12.4 (11.1)	5.8 (6.4)
HAQ (0-3)^a^	1.2 (0.8)	0.9 (0.7)	0.7 (0.6)	0.7 (0.7)
AQoL (0-1)^a^	0.5 (0.2)	0.6 (0.2)	0.6 (0.2)	0.6 (0.3)
EQ-5D (UK) (0-1)^a^	0.6 (0.3)	0.6 (0.3)	0.6 (0.3)	0.7 (0.2)
SF-36 PCS (0-100)^a^	31.4 (10.7)	35.1 (11.1)	38.2 (10.9)	41.4 (11.3)
SF-36 MCS (0-100)^a^	45.5 (12.0)	46.4 (11.8)	46.0 (11.1)	50.3 (9.6)
Pain in last week (0-100)^a^	47.4 (26.4)	45.3 (25.9)	39.5 (27.9)	31.8 (27.9)
Arthritis condition in last week (0-100)^a^	45.2 (26.2)	45.0 (26.7)	39.9 (26.6)	40.2 (26.2)
	N (%)	N (%)	N (%)	N (%)
Female	2,911 (73.5)	446 (59.5)	247 (33.6)	128 (69.2)
Education				
Not completed secondary	1,096 (28.9)	158 (21.1)	124 (17.2)	133 (79.6)
Secondary	1,277 (33.6)	204 (27.3)	199 (27.6)	21 (12.6)
Tertiary	1,424 (37.5)	386 (51.6)	397 (55.1)	13 (7.8)
Employment				
Working	1,415 (37.2)	413 (55.2)	482 (67.1)	24 (14.6)
Not working/home duties/student/retired/other	1,907 (50.1)	267 (35.7)	184 (25.6)	139 (84.8)
Permanently unable/ill	485 (12.7)	68 (9.1)	52 (7.2)	1 (0.6)
Socioeconomic index				
SEIFA 1 lowest	580 (15.0)	90 (12.5)	76 (10.7)	25 (13.8)
SEIFA 2	726 (18.8)	126 (17.5)	114 (16.0)	24 (13.3)
SEIFA 3	788 (20.4)	144 (20.0)	150 (21.1)	42 (23.2)
SEIFA 4	891 (23.1)	169 (23.5)	165 (23.2)	38 (21.0)
SEIFA 5 highest	877 (22.7)	191 (26.5)	206 (29.0)	52 (28.7)
Current private health insurance	2,182 (55.1)	489 (65.3)	443 (60.2)	101 (54.6)
Current smoker	544 (13.8)	87 (11.6)	121 (16.4)	4 (2.4)
Alcohol use				
Never	1,455 (36.8)	143 (19.1)	144 (19.6)	137 (82.0)
Sometimes	2,040 (51.5)	499 (66.6)	484 (65.8)	30 (18.0)
Everyday	464 (11.7)	107 (14.3)	108 (14.7)	0 (0.0)
Current opioid use (potency)				
No	2678 (67.6%)	529 (70.6%)	525 (71.3%)	166 (89.7%)
Yes (low)	861 (21.7%)	129 (17.2%)	157 (21.3%)	15 (8.1%)
Yes (high)	421 (10.6%)	91 (12.1%)	54 (7.3%)	4 (2.2%)
Current NSAID use				
No	2409 (60.8%)	360 (48.1%)	364 (49.5%)	118 (63.8%)
Yes	1551 (39.2%)	389 (51.9%)	372 (50.5%)	67 (36.2%)
Current b/tsDMARD	2,125 (53.7)	377 (50.3)	532 (72.3)	99 (53.5)
Prednisolone^b^				
Current	1,756 (44.3)	179 (23.9)	77 (10.5)	44 (23.8)
Past	818 (20.7)	225 (30.0)	214 (29.1)	72 (38.9)
Methotrexate^b^				
Current	2,463 (62.2)	424 (56.6)	95 (12.9)	123 (66.5)
Past	732 (18.5)	259 (34.6)	156 (21.2)	44 (23.8)
Number of complementary medicines				
0	2,458 (62.1)	415 (55.4)	455 (61.8)	146 (78.9)
1	854 (21.6)	198 (26.4)	160 (21.7)	26 (14.1)
2	426 (10.8)	81 (10.8)	80 (10.9)	10 (5.4)
≥3	222 (5.6)	55 (7.3)	41 (5.6)	3 (1.6)

^a^Higher score = poorer function, ^b^Balance never or do not know. HAQ: Health Assessment Questionnaire; AQoL: Assessment of Quality of Life; EQ-5D (UK): European Quality of Life; SF-36 PCS: Medical Outcomes Survey Short Form—physical component score; SF-36 MCS: Medical Outcomes Survey Short Form—mental component score; SEIFA: Socio-Economic Indexes for Areas; NSAID: nonsteroidal anti-inflammatory drug; b/tsDMARD: biologic/targeted synthetic disease-modifying antirheumatic drug.

**Table 2:** CM use at baseline across all ARAD participants.

Fatty acids (n) *N* = 1,564	Herbal medicine (n) *N* = 296	Supplements (n) *N* = 808
Fish oil (1,489)	Ginger (148)	Glucosamine (605)
NZ green lipped mussels (53)	Celery seed (42)	Multivitamins (55)
Evening primrose oil (21)	Chinese herbs (40)	Magnesium (45)
Flaxseed oil (19)	St John's wort (23)	Shark fin (28)
Krill oil (19)	Kelp (24)	Vitamin C (28)
Olive oil (11)	Turmeric/cumin/curcumin (19)	Vitamin B (25)
Emu oil (9)	Pennywort (14)	Vitamin E (17)
Cod liver oil (9)	Garlic (15)	Probiotic (17)
Linseed oil (4)	Rosehip (10)	Zinc (16)
Hemp oil (1)	Cranberry (1)/fruit juice (7)	Chondroitin (11)
	Gingko (7)	Coenzyme Q10 (5)
	Milk thistle (6)	Silver (2)
	Lysine (4)	
	Spirulina (4)	
	Apple cider vinegar (4)	
	Chia tea (2)	
	Bee pollen (1)	
	Aloe vera (1)	
	Barley (1)	
	Cat's claw (1)	
	Devil's claw (1)	
	Echinacea (1)	
	Gotu (1)	
	Green tea (1)	

**Table 3:** Factors associated with complementary medicine use compared to no CM use, univariate, and multivariable analysis.

Variable	All CM univariate	All CM multivariable
	Odds ratio (95% confidence interval)	Odds ratio (95% confidence interval)
Disease		
RA (*N* = 3960)	1.00	1.00
PsA (*N* = 749)	1.32 (1.13-1.55)	0.97 (0.82-1.15)
AS (*N* = 736)	1.04 (0.89-1.23)	1.02 (0.84-1.25)
JIA (*N* = 185)	0.37 (0.27-0.58)	0.44 (0.25-0.75)
Greater disease duration	0.99 (0.98-1.00)	0.99 (0.98-0.99)
Increased age	1.01 (1.01-1.01)	1.01 (1.01-1.02)
Sex		
Male	1.00	1.00
Female	1.37 (1.22-1.53)	1.30 (1.13-1.50)
Function (HAQ)^a^	0.89 (0.82-0.95)	1.13 (1.02-1.24)
Education		
Not completed secondary	1.00	1.00
Secondary	0.88 (0.76-1.02)	1.11 (0.94-1.32)
Tertiary	1.29 (1.13-1.47)	1.32 (1.13-1.55)
Private health insurance		
No	1.00	1.00
Yes	1.35 (1.20-1.51)	1.26 (1.10-1.44)
Current smoking		
No	1.00	1.00
Yes	0.81 (0.56-0.63)	0.76 (0.63-0.91)
Alcohol		
Never	1.00	1.00
Sometimes	2.44 (2.15-2.78)	1.22 (1.05-1.43)
Everyday	2.06 (1.71-2.48)	1.03 (0.83-1.28)
Opioid use		
No	1.00	1.00
Low potency	1.82 (1.60-2.09)	1.21 (1.05-1.41)
High potency	1.62 (1.35-1.93)	1.08 (0.89-1.32)
NSAID use		
No	1.00	1.00
Yes	2.02 (1.81-2.25)	1.32 (1.17-1.50)
Employment		
Working	1.00	
Not working	0.95 (0.85-1.07)	
Permanently unable/ill	0.78 (0.64-0.94)	
Socioeconomic index		
SEIFA 1 lowest	1.00	
SEIFA 2	1.09 (0.90-1.33)	
SEIFA 3	1.12 (0.92-1.36)	
SEIFA 4	1.20 (0.99-1.44)	
SEIFA 5 highest	1.27 (1.05-1.53)	
Current bDMARD		
No	1.00	
Yes	1.11 (1.00-1.24)	
Quality of life (AQoL)^b^	1.37 (1.10-1.71)	
Quality of life (EQ-5D)^b^	1.56 (1.27-1.91)	
Physical health (SF-36)^c^	1.00 (1.00-1.01)	
Mental health (SF-36)^c^	1.00 (1.00-1.01)	
Pain past week^c^	1.00 (1.00-1.00)	
Overall arthritis activity past week^c^	1.00 (1.00-1.00)	

HAQ: Health Assessment Questionnaire; AQoL: Assessment of Quality of Life; EQ-5D (UK): European Quality of Life; SF-36 PCS: Medical Outcomes Survey Short Form—physical component score; SF-36 MCS: Medical Outcomes Survey Short Form—mental component score; SEIFA: Socio-Economic Indexes for Areas; NSAID: Nonsteroidal anti-inflammatory drug; b/tsDMARD: biologic/targeted synthetic disease-modifying antirheumatic drug. ^a^Range 0–3, where higher scores indicate greater functional impairment. ^b^Range 0–1, where higher scores indicate lower quality of life. ^c^Range 0–100, where higher scores indicate greater impairment.

## Data Availability

The datasets used and/or analysed during the current study are available from the corresponding author on reasonable request.
